# Robot‐Assisted Distal Pancreatectomy Using the Hugo RAS System via the Supragastric Approach: First Nationwide Case Report (With Video)

**DOI:** 10.1111/ases.70239

**Published:** 2026-01-14

**Authors:** Kazuyuki Nagai, Kei Yamane, Asahi Sato, Hiroto Nishino, Takahiro Nishio, Katsunori Sakamoto, Satoshi Ogiso, Yoichiro Uchida, Takashi Ito, Takamichi Ishii, Shigeo Hisamori, Kazutaka Obama, Etsuro Hatano

**Affiliations:** ^1^ Division of Hepato‐Biliary‐Pancreatic Surgery and Transplantation, Department of Surgery, Graduate School of Medicine Kyoto University Kyoto Japan; ^2^ Division of Gastrointestinal Surgery, Department of Surgery, Graduate School of Medicine Kyoto University Kyoto Japan

**Keywords:** first case, Hugo RAS system, robotic distal pancreatectomy, supragastric approach

## Abstract

The Hugo RAS System received regulatory approval for gastroenterological surgery in Japan in May 2023. Beyond standard robotic advantages—three‐dimensional high‐definition imaging, articulated instruments with tremor filtration, and motion scaling—it features an open console that enhances team communication and four independent arm carts for flexible positioning. We report Japan's first case of robot‐assisted distal pancreatectomy performed using the Hugo RAS System. The patient was a 78‐year‐old woman with a branch‐duct intraductal papillary mucinous neoplasm in the left pancreas exhibiting high‐risk stigmata. Four ports were placed laterally at the umbilical level, with an assistant port in the right subcostal region. Arm cart 1 was positioned on the patient's right caudal side, and the remaining three on the left cranial side. After division of the splenic artery via the supragastric approach, the pancreas and spleen were mobilized, and transection was performed at the pancreatic neck for specimen retrieval.

## 1 Introduction

Robot‐assisted distal pancreatectomy (RDP) has been increasingly adopted worldwide in recent years for left‐sided pancreatic tumors [1]. Minimally invasive distal pancreatectomy, including RDP, reduces blood loss and shortens hospitalization compared with open surgery [2, 3], while its long‐term oncological outcomes for pancreatic cancer, although not yet confirmed in randomized controlled trials, appear comparable [3, 4].

In Japan, several robotic platforms are available, each with distinct features despite similar core functions. Among them, the da Vinci Surgical System (Intuitive Surgical Inc., Sunnyvale, CA, USA) and Hinotori Surgical Robot System (Medicaroid Corporation, Kobe, Japan) [5] have been predominantly used [6]. The Hugo RAS System (Medtronic, Minneapolis, MN, USA), featuring its open‐console design and four independently movable arm carts, received regulatory approval from the Japanese Ministry of Health, Labour, and Welfare for gastroenterological surgery in May 2023. Following nationwide case accumulation in gastrointestinal procedures confirming its safety, the author was certified by the Japanese Society of Hepato‐Biliary‐Pancreatic Surgery as the “first surgeon” authorized to perform RDP using this system in September 2025 and subsequently initiated the procedure.

We herein report the first RDP using the Hugo RAS System performed in Japan, which, to our knowledge, is also the first case reported worldwide.

## 2 Case Presentation

**FIGURE 1:**
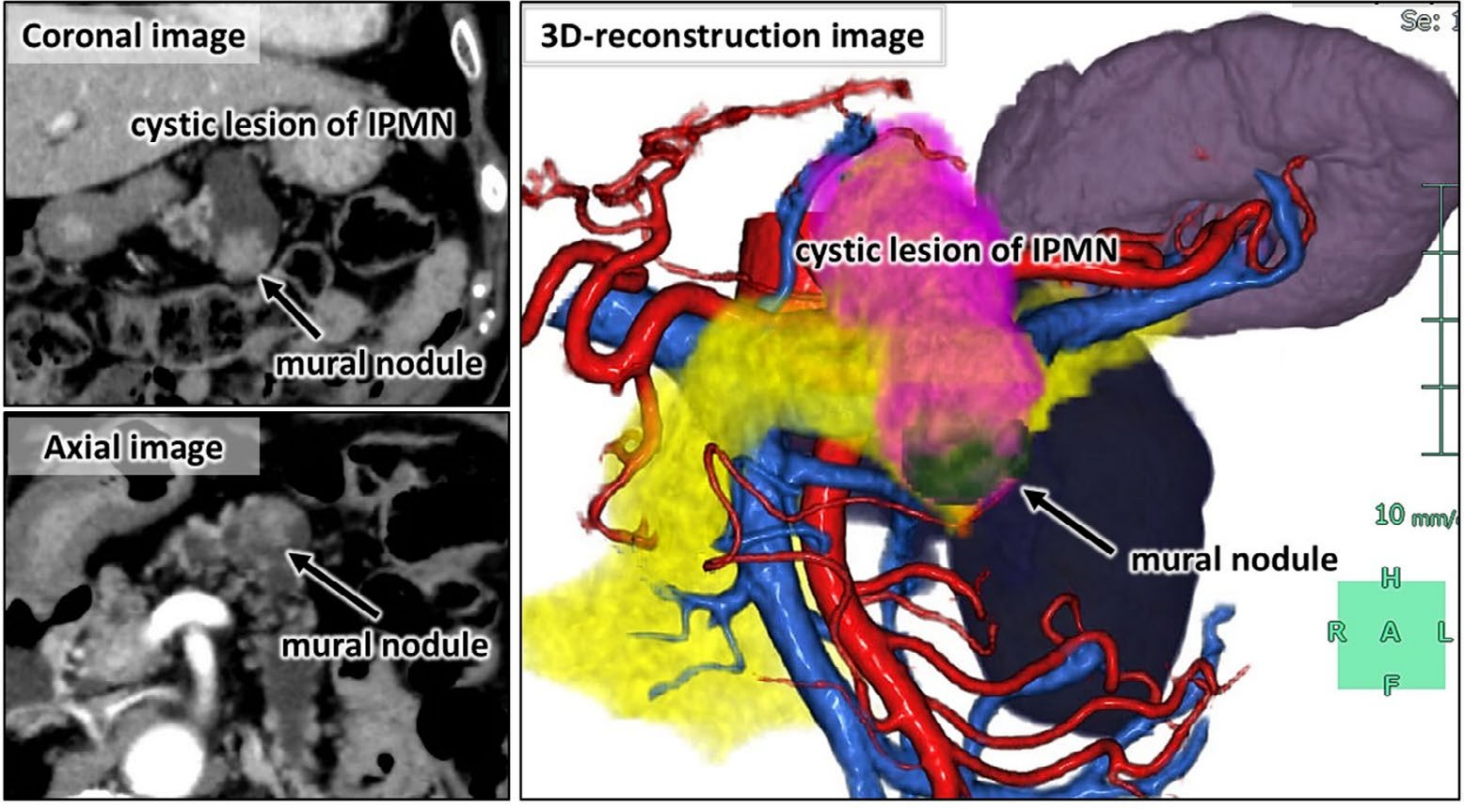
Coronal and axial CT images, along with a three‐dimensional reconstructed image, showing a 60‐mm branch‐duct intraductal papillary mucinous neoplasm (IPMN) in the pancreatic body and tail with a 16‐mm enhancing mural nodule.

The patient was a 78‐year‐old woman referred to our hospital for evaluation of a progressively enlarging pancreatic cyst. Computed tomography revealed a 60‐mm cystic lesion in the pancreatic body and tail, communicating with the main pancreatic duct and containing a mural nodule (Figure 1). Contrast‐enhanced endoscopic ultrasonography showed a 16‐mm enhancing mural nodule and main duct dilation to 8.6 mm. The lesion was diagnosed as a branch‐duct intraductal papillary mucinous neoplasm (IPMN) with high‐risk stigmata, and RDP with lymphadenectomy and splenectomy was performed using the Hugo RAS System.

**FIGURE 2:**
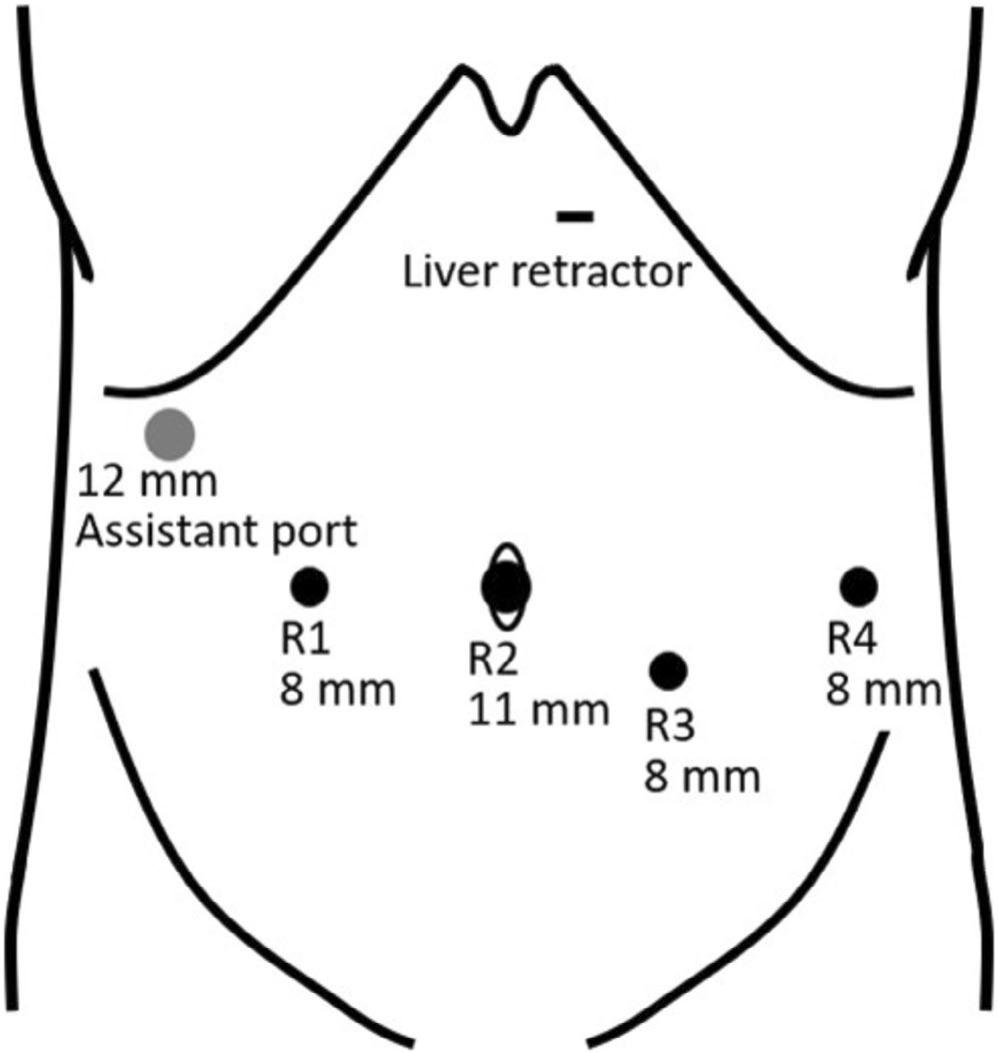
Port placement. R1–R4 indicate the robotic arms (R1, R3, R4: 8‐mm ports; R2 for the videoscope: 11‐mm port). The assistant port was placed below the right costal arch along the anterior axillary line, and the Nathanson liver retractor was positioned in the epigastric region.

**FIGURE 3:**
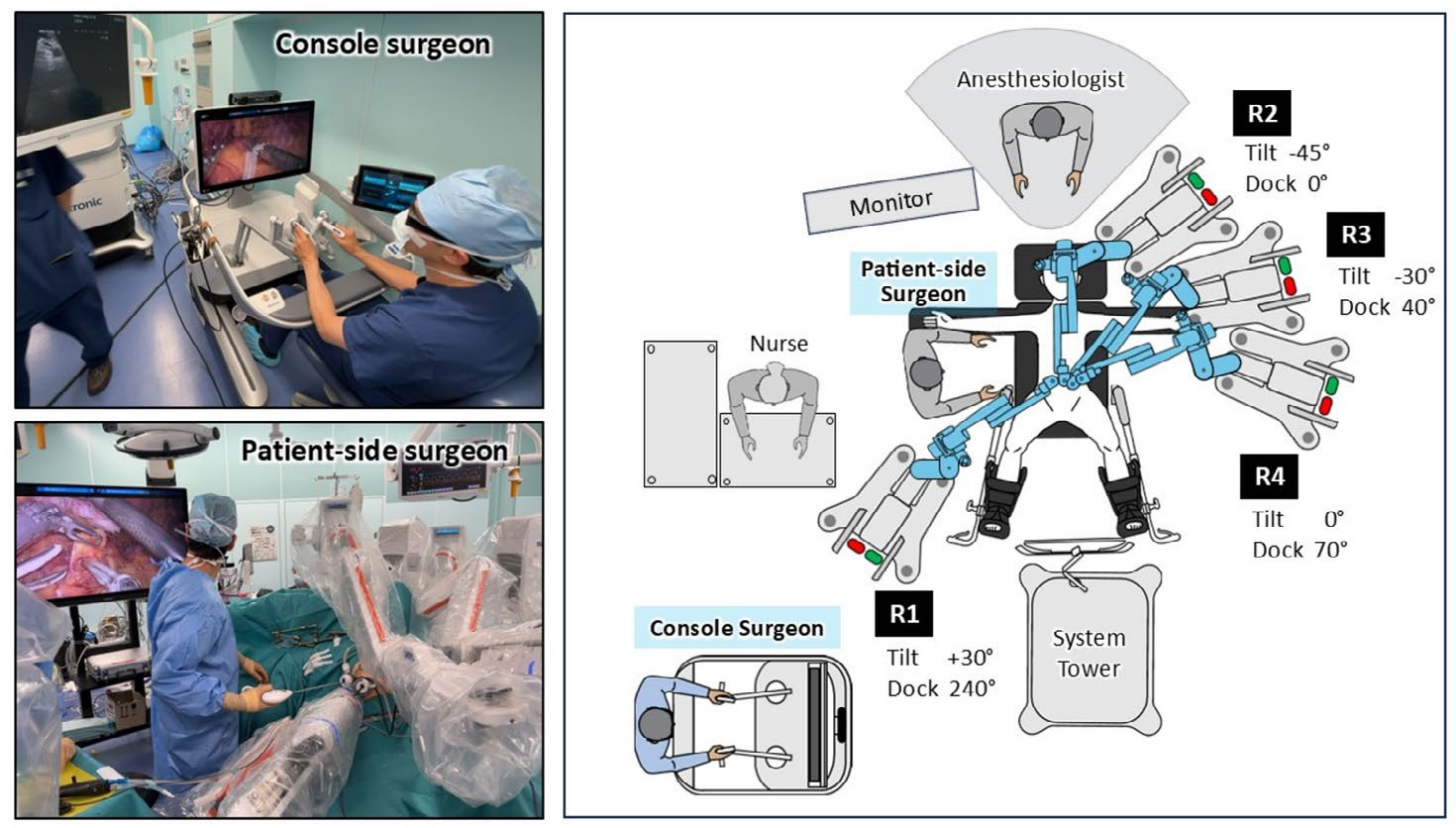
Operating room setup and the Hugo RAS System. Arm carts R2–R4 were positioned on the patient's left side in sequence from the cranial side, while arm cart R1 was placed on the right caudal side. The console surgeon operated from the open console, and the patient‐side surgeon was positioned on the patient's right.

The patient was placed in the lithotomy position using a levitator. After port insertion, the table was tilted 13° head‐up and 7° left‐side‐up. Port placement and room setup are shown in Figures 2 and 3. A 11‐mm port for the second robotic arm (R2; Hugo Optical Trocar 11 mm STF, Medtronic) was inserted at the umbilicus for a 30° videoscope. The first arm (R1) port was 7 cm right of the umbilicus, the fourth (R4) 14 cm left, and the third (R3) 3 cm caudal to the midpoint between R2 and R4. Ports R1, R3, and R4 used 8‐mm trocars (Hugo Universal Cannula 8 mm, Medtronic). A 12‐mm assistant port (AirSeal Access Port, CONMED, NY, USA) was placed below the right costal arch on the anterior axillary line. Arm cart 1 was on the patient's right caudal side, and the remaining three on the left cranial side. Arms R1 and R4 carried Cadiere forceps, and R3 bipolar Maryland forceps connected to the Valleylab FT10 energy platform (Medtronic, Macro mode, 60 W). The patient‐side surgeon used a LigaSure Maryland jaw device (44 cm, Medtronic) for vessel sealing, suction, and gauze handling.

**FIGURE 4:**
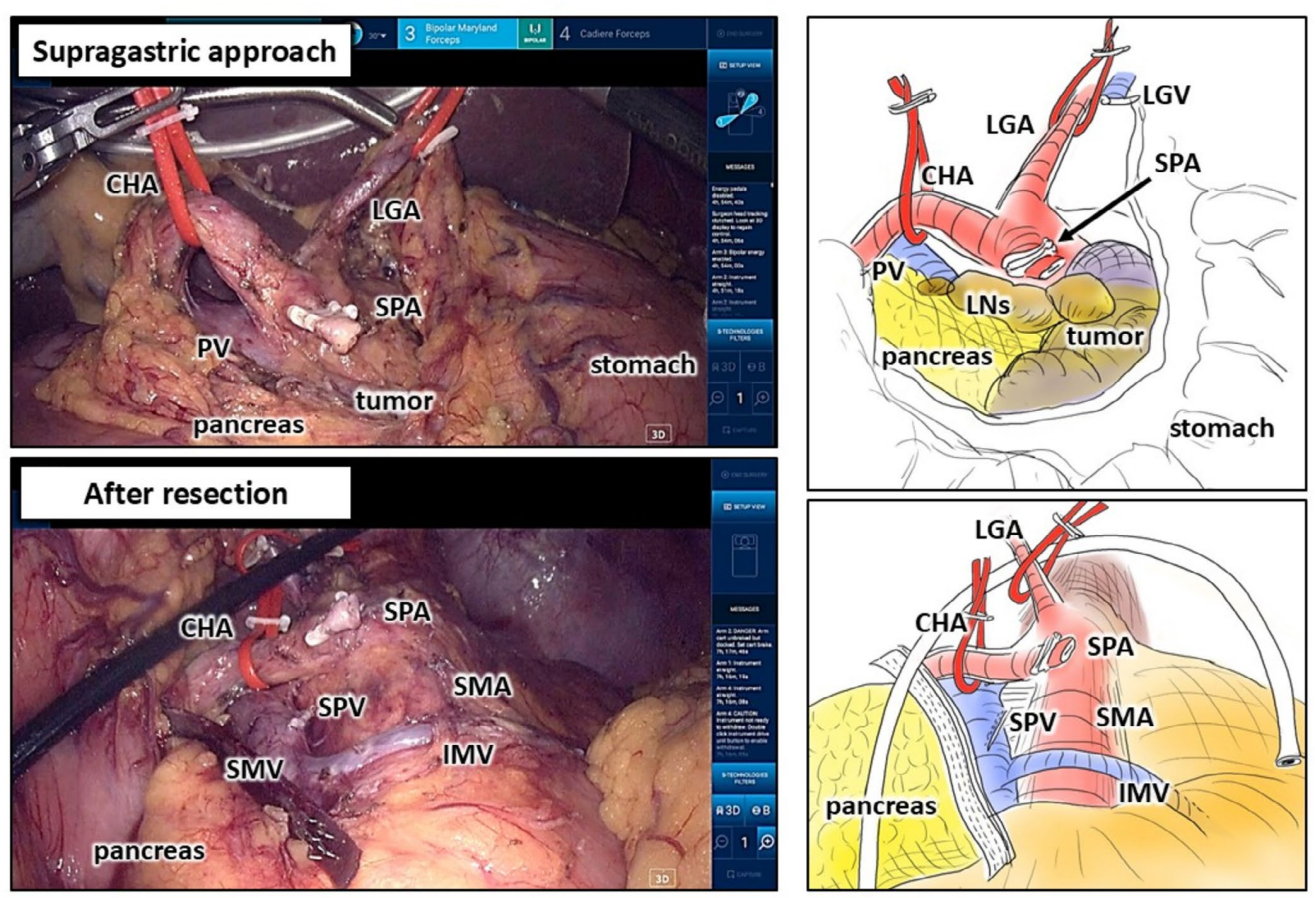
Intraoperative views after the supragastric approach and pancreatic resection. Resection was performed along anatomical landmarks. CHA, common hepatic artery; IMV, inferior mesenteric vein; LGA, left gastric artery; LGV, left gastric vein; LNs, lymph nodes; PV, portal vein; SMA, superior mesenteric artery; SPA, splenic artery; SPV, splenic vein.

Laparoscopically, the omental bursa was opened and the splenocolic ligament divided (Video S1). The Hugo RAS System was then docked, and robotic procedures initiated using a “supragastric approach,” in which dissection proceeds from the lesser curvature of the stomach to secure an optimal operative field without interference from gastric retraction (Figure 4). After opening the lesser omentum, the anterior surface of the diaphragmatic crus was dissected. The left gastric vein was divided, and the left gastric artery identified. Lymph node station #8 [7] was dissected en bloc with the pancreas, exposing the splenic artery root. After clipping and dividing the splenic artery, dissection proceeded along the posterior gastric wall toward the splenic upper pole and along Gerota's fascia behind station #11 [7]. The posterior gastric wall was elevated using traction sutures and a Nathanson retractor. The pancreas and spleen were mobilized and connected to the plane created by the supragastric approach, followed by dissection along the anterior surface of the superior mesenteric artery. The splenic vein was divided with a stapler (Signia Small Diameter Reload, 30 mm, white cartridge, Medtronic), and the pancreas taped. The gastrosplenic ligament was divided, leaving the specimen attached only at the pancreas. Intraoperative ultrasonography confirmed the tumor location and transection line. R3 was replaced with a 15‐mm port (VersaOne Optical Trocar 15 mm, Medtronic), and the pancreatic neck divided using a linear stapler (Signia, 60 mm, black cartridge with reinforcement, Medtronic). The specimen was retrieved through the umbilical incision. After irrigation and drain placement, the procedure was completed (Figure 4).

The operative time was 362 min, with a console time of 228 min and estimated blood loss of 15 mL. No postoperative complications of Clavien–Dindo grade IIIa or higher, including pancreatic fistula, occurred, and the patient was discharged on postoperative Day 12. Pathological examination revealed a branch‐duct IPMN (gastric type) with high‐grade dysplasia. Thirty lymph nodes were dissected, and R0 resection was achieved.

## 3 Discussion

Several robotic surgical systems are available worldwide. In Japan, the main platforms for pancreatic resection are the da Vinci Surgical System and the Hinotori Surgical Robot System [6]. Although these systems share core features—three‐dimensional high‐definition imaging, articulated instruments with tremor filtration, and motion scaling—each has unique characteristics that must be well understood.

The Hugo RAS System is distinguished by its open‐console design, which facilitates team communication, and four independently movable arm carts offering greater flexibility in positioning than conventional integrated‐arm systems [8]. However, vessel‐sealing devices, clips, and staplers are not yet available as robotic instruments; therefore, these tasks must be performed by the patient‐side surgeon using conventional laparoscopic tools. Optimal port placement and arm configuration are critical for efficient assistance. In our setup, the R1 arm cart was positioned on the patient's caudal side, and the patient‐side surgeon's port was placed lateral and cranial to R1, ensuring adequate working space. Another limitation is that the double‐bipolar method [9, 10], commonly used in robotic surgery in Japan, cannot be employed. We used bipolar forceps in the right hand for precise dissection and division, while the left‐hand bipolar for soft coagulation was omitted. Despite this, the operation proceeded smoothly with active assistance from the patient‐side surgeon.

In the present case, several surgical steps were intentionally performed laparoscopically prior to robotic docking for two reasons. First, the omental bursa was opened at an early stage to allow for the initial inspection of the lesion. Second, the mobilization of the splenic flexure, which involves deep operative maneuvers, was performed in advance, as this approach was considered potentially safer for this initial experience with the system.

Reducing operative time is a crucial concern in robotic surgery. In this case, the docking process, defined as the interval from initiation of roll‐in to the start of console manipulation, was completed within 20 min, consistent with the preoperative simulation. Although several laparoscopic steps were performed before and after the robotic manipulation, such as the aforementioned pre‐docking procedures, intraperitoneal lavage, and drain placement after roll‐out, there is potential for further refinement of the workflow to enhance procedural efficiency in future cases.

Robotic surgery is increasingly being applied to complex pancreatic procedures, including pancreatoduodenectomy. Because these operations require a wide intra‐abdominal surgical field, the flexible arm configuration of the Hugo RAS system may offer a particular advantage. Moreover, given the relatively long operative times associated with such procedures, the open‐console design may help maintain a more natural surgeon posture, potentially reducing surgeon fatigue. Nonetheless, for complex pancreatic surgery, the development and availability of advanced robotic instruments, such as a double‐bipolar method, articulating vessel‐sealing devices, clips, and staplers, would enhance the operative capabilities of the console surgeon.

We successfully performed the first RDP using the Hugo RAS System in Japan, which, to our knowledge, also represents the first reported case worldwide. Further case accumulation is needed to confirm its safety, and ongoing technological advances are expected to broaden its future application.

## Author Contributions

All authors made substantial contributions to the study and agree with the content of the article.

## Funding

The authors have nothing to report.

## Ethics Statement

All the procedures were performed in accordance with the principles of the Declaration of Helsinki. This study was approved by the Ethics Committee of Kyoto University (R5212).

## Consent

Informed consent was obtained from the patients for the publication of this case report. The patient provided written informed consent to participate in this study.

## Conflicts of Interest

Dr. Kazutaka Obama is an Editorial Board member of ASES Journal and a co‐author of this article. To minimize bias, they were excluded from all editorial decision‐making related to the acceptance of this article for publication. The other authors declare no conflicts of interest.

## Supporting information


**Video S1:** Robot‐assisted distal pancreatectomy using the Hugo RAS System. After division of the splenic artery via the supragastric approach, the pancreas and spleen were mobilized, and transection was performed at the pancreatic neck for specimen retrieval.

## Data Availability

The data that support the findings of this study are available from the corresponding author upon reasonable request.
